# “Liberty Can Be for You One Thing, and for Me Something Different”: Muslim Women's Experiences of Identity and Belonging in Switzerland

**DOI:** 10.5964/ejop.10623

**Published:** 2023-11-30

**Authors:** Rachael Loxston, Liza Jachens

**Affiliations:** 1 Psychology, Sociology and Professional Counselling, Webster University, Geneva, Switzerland; 2 Centre for Organizational Health and Development, Division of Psychiatry and Applied Psychology, School of Medicine, University of Nottingham, United Kingdom; Victoria University of Wellington, Wellington, New Zealand

**Keywords:** counseling, Muslim women, gendered Islamophobia, identity, belonging, Switzerland

## Abstract

Belonging and identity are fundamental human needs, with positive experiences closely correlated with affirmative mental health. This paper investigates how these concepts are experienced by Muslim women in Switzerland, a minority group targeted in the political campaign nicknamed the “burka ban.” There were two research questions: How do Muslim women construct their identity in Switzerland? How do Muslim women experience a sense of belonging in Switzerland? Semi-structured interviews were conducted with six participants, and data was analyzed using thematic analysis. Six themes were identified: religion as a public versus private identity, Islam and dressing modestly as expressions of gendered liberation, sharing a sense of Swiss identity through sameness, challenging dominant representations, impression management, and religious and cultural identity as psychological strengths. Participants used several strategies to construct a positive identity and experience belonging in response to negative representation. Findings are summarized in the form of recommendations for counselors working in Switzerland.

In recent years, there has been a renewed interest in Islam and its place in European society (Grinell, 2020). Specifically, there has been a collective fixation on Muslim women’s dress, bringing Muslim women to the political forefront. Around one-third of European countries have introduced some form of national or local legislation prohibiting Islamic veiling (Abdelgadir & Fouka, 2020). The broader media and political focus on Islam and Muslim women’s attire contribute to gendered Islamophobia (Allen, 2015) and have encouraged the view that Muslim women have become *the* symbol of Islam in Western countries (Chakraborti & Zempi, 2012).

## Gendered Islamophobia

In its seminal report, the Runnymede Trust (1997) defines Islamophobia in three parts: first as “unfounded hostility towards Islam,” second as “the practical consequences of such hostility in unfair discrimination against Muslim individuals and communities,” and third as “the exclusion of Muslims from mainstream political and social affairs” (Runnymede Trust, 1997, p. 4). Importantly, Islamophobic attacks in Western countries consistently target Muslim women (Zempi, 2020). Several factors contribute to this phenomenon, including the intersection of multiple identities, such as race, religion, and gender. Crenshaw (1991) coined the term *intersectionality* to highlight how different identities intersect to contribute to an individual’s unique experiences of discrimination. Muslim women who choose to veil are particularly vulnerable, as their dress makes them a visible target for Islamophobes (Allen, 2015).

## Consequences of Anti-Muslim Sentiment

Muslim women in Europe are at risk of suffering physical or verbal attacks, ethnic and religious profiling, and restrictions on religious practices (Open Society Foundations, 2019). The psychological impact of discrimination can be significant since an individual’s identity is formed partially through recognition by others, including their perception of how the groups to which they feel they belong are recognized (Taylor, 1994). Cohen and Garcia (2005) argue that negative group representation and recognition can encourage fear in in-group members, known as a collective threat. The term *collective threat* encapsulates group members’ desire to be viewed positively and the undue stress this creates for them as they carry the responsibility of defying group stereotypes.

The psychological burden of representing an entire religion and the threat that negative group recognition poses to an individual’s sense of identity and belonging have also been demonstrated in studies on hate crimes. Research has revealed that, regardless of their severity, hate crimes can cause severe psychological distress because victims are targeted due to an important aspect of their identity (Iganski & Lagou, 2014). A victim’s awareness of being singled out for who they are and what they represent can have a lasting impact on them and their community (Chakraborti & Garland, 2012).

Concealing an identity is a strategy often employed after a threat (Sønderlund et al., 2017). However, this strategy has been found to negatively affect well-being. In addition, disruptions to an individual’s sense of belonging can impair cognitive, emotional, and physiological functioning (Andersen et al., 2000) and increase mental health disorders (Victim Support, 2013). This renders the study of identity and belonging critical for mental health professionals.

## Identity and Sense of Belonging

For this paper, *identity* is defined as an essential aspect of the self (individual and collective) and central to people’s understanding of themselves as social beings. This definition is understood as “something to be valued, cultivated, supported, recognized, and preserved” (Brubaker & Cooper, 2000, p. 7). On a collective level, identity is established through recognizing similarity, either objectively through shared characteristics or subjectively through imagined collective experiences.

Belonging is a fundamental human need (Andersen et al., 2000; Maslow, 2019) and is a measure of an individual’s connection to their social and physical environments (Hill, 2006). This research adopts the two dimensions of a sense of belonging that Hagerty et al. (1992) proposed. First, a sense of belonging is constructed through “valued involvement,” the “experience of feeling valued, needed, [and] accepted” (p. 173). Second, through “fit,” the individual must perceive that their “characteristics articulate with or complement the system or environment” (p. 173).

## Muslim Women in Europe

Identity and belonging have emerged as significant themes in studies that explore the experiences of Muslim women in Europe (Allen, 2015; Bouteldja, 2011; van Es, 2019). These studies were conducted during periods of heightened anti-Islamic discourse. Allen (2015) found that veiled Muslim women in the UK suffered discrimination based on being representatives of Islam. Participants reported being cast as unwelcome outsiders regardless of additional identifying markers, such as being a British national.

Disruptions to a sense of belonging were found by Bouteldja (2011) who documented the experiences of 32 veiled Muslim women before and after the French national banning of the face veil. Several findings support the view that Muslim women sought to assert their religious identity in reaction to increased scrutiny (Bouteldja, 2011; El-Halawany, 2003; Gupta, 2004; Kundnani, 2002; van Es, 2019). Van Es (2019) highlighted that Muslim women reacted to scrutiny by becoming self-appointed ambassadors of Islam and representing themselves as “modern” and “emancipated” (van Es, 2019, p. 389), for some women wearing a headscarf allowed them to be “visibly Muslim,” which was important in constructing their ambassador role.

As Muslim women are aware of stereotypes about them and yet are unaware of which individuals hold these views, they monitor their behavior. Women, therefore, participate in essentializing their Muslim identity by “reducing their own identity to a single element” (van Es, 2019, p. 387). This reduction in identity may negatively affect individual well-being, particularly when the salient identity is threatened (Barnett & Hyde, 2001; Koch & Shepperd, 2004).

## Muslim Women in Switzerland

Despite the increasing number of academic papers on Islamophobia in Europe, research in Switzerland is limited (Yendell & Huber, 2020). The Muslim minority accounts for only around 5% of the Swiss population (Schweizerische Eidgenossenschaft Confédération Suisse, 2021a), yet Islam in Switzerland is strongly debated (Arlt, 2021).

On March 7, 2021, Switzerland introduced a ban on face coverings in public (BBC, 2021, March 7). Although the ban includes all items of clothing that cover an individual’s face, it was dubbed the “burka ban” due to the focus on Islamic dress. Provocative posters of menacing-looking Muslim women alongside slogans such as “Stop extremism” were displayed across the country (Waterfield, 2021).

Despite this focus on Muslim women in Switzerland, little is known about their experiences, so further exploration is recommended (Gasser, 2020)^1^1Entire article translated from German into English using DeepL Pro software.. The need for further research becomes apparent when one considers aspects that separate Switzerland from neighboring countries. Switzerland’s immigration and citizenship policies are considered some of the strictest in Europe, and its migration policies seem designed to encourage assimilation (Gasser, 2020). Additionally, Switzerland’s Muslim population is more heterogeneous than neighboring countries such as France and Germany.

Furthermore, events that spotlight Muslims can increase Islamophobic incidents (Allen, 2005; Bouteldja, 2011). The “burka ban” has been a catalyst for ongoing debates on Muslim women’s dress in Switzerland—for example, the relevance of the headscarf in schools and the appropriateness of the burkini in public swimming pools (Gasser, 2020)^2^2Entire article translated from German into English using DeepL Pro software.. Under these restrictions, Muslim women are confined to “the conditions of European stereotypical gender construction” and denied the freedom to choose what they wear (Kaya, 2012, p. 124)^3^3Entire article translated from German into English using DeepL Pro software..

## Research Aims and Questions

Two research questions were posed. First, how do Muslim women construct their identity in Switzerland? This question is used to assess how Muslim women make sense of the political and social narratives constructed about them and express their Muslim identity. Second, how do Muslim women experience belonging in Switzerland? Given the political campaigns that have singled out Muslim women and argued that certain Islamic symbols do not belong in Switzerland, this question is used to establish how Muslim women have experienced belonging since the “burka ban.” This research also aims to aid counselors in clinical practice in Switzerland by highlighting the plight of a minority group. The interviews and research in this investigation are intended to identify coping mechanisms that the population might use.

## Method

### Research Design

A qualitative method was chosen so participants could express their unique experiences. Participants’ experiences were collected using semi-structured, in-depth individual interviews. The study received ethical approval from the Institutional Review Board of Webster University.

### Participant Selection

Various methods were used to recruit participants, including asking the first author’s network of friends and acquaintances, attending a one-day networking event for young Swiss Muslims (which led to the opportunity to recruit through a trusted community member), and posting a flyer on several Facebook groups for English speakers in Switzerland. Recruiting participants using different approaches reflected the challenge of finding participants. Furthermore, researchers who are considered outsiders often experience more significant difficulties with recruitment than those who are considered in-group members (Okumus et al., 2007).

We sought women over 18 years of age who self-identified as Muslim. In addition, participants were required to be current Swiss nationals or permanent residents (short-term visas excluded). The decision to include residents and nationals was due to the lengthy process of applying for Swiss citizenship (Schweizerische Eidgenossenschaft Confédération Suisse, 2021b). The interviews were conducted in English; therefore, participants were required to be comfortable answering in-depth questions in this language. All participants provided both their written and verbal informed consent.

### Sample Description

The sample consisted of six Muslim women currently living and working in Switzerland. Participants were assigned Arabic pseudonyms to ensure anonymity. The women interviewed varied in nationality, age, ethnicity, location, languages spoken, and length of stay in Switzerland (see Table 1). Additionally, three of the participants wore the hijab, and three did not.

**Table 1:** Participants Demographics

Age	Nationality	Ethnicity	Languages Spoken	Length of Stay	Canton	Wore Hijab	Pseudonym
42	Singaporean	Indian	English, Malay, Urdu	3 years	Zürich	No	Faheema
58	Swiss/Sri-Lankan	Ceylon Moors	English, French	32 years	Geneva	No	Hind
36	Swiss	Pakistani	English, Italian, Urdu, German, French	36 years	Ticino & Zürich	Yes	Nur
29	Swiss	Somali	English, German, Swiss German, Somali	28 years	Zürich	Yes	Amira
30	Swiss	European	English, German, Swiss German, Spanish, Catalan, French	6 years in total; 3 years at a time	Zürich	Yes	Zaara
49	British	British-Pakistani	English, Urdu, Arabic, French, Punjabi	4 years	Vaud	No	Fara

*Note.* The nationality and ethnicity descriptions reflect how the participants described themselves.

### Data Collection: Qualitative Interviews

The qualitative interviews were conducted via Zoom by the first author (camera on by choice). Individual interviews were conducted to encourage disclosure and limit external influences.

The semi-structured format and interview questions were guided by previous research (Moradi et al., 2019; Sadiq, 2019). The interview guide was used to encourage exploration of experiences of belonging and identity and included questions such as the following:

1. What details about yourself and how you live identify you as a Muslim woman?
2. Would you tell me something about your experience as a Muslim woman in Switzerland?
3. In what way(s) do you feel Switzerland is your home?

The first author utilized follow-up questions, reflective listening, and summarizing to confirm understanding and identify coping mechanisms. The interviews lasted 56 minutes on average, with the shortest lasting 40 minutes and the longest 90 minutes.

### Analysis

The interview transcripts were analyzed using Braun and Clarke’s (2006) six-phase guide to thematic analysis using an inductive approach. Two coders (authors) coded the material separately. Codes were generated and collated into prospective themes that were then reviewed and named. After this step, the coders worked together to expose disagreements and reach a consensus on the final themes.

After the final themes and accompanying quotations were chosen, the participants were sent the analysis via email and invited to comment on the interpretation of their experiences with written feedback. Four of the six participants participated in this step and confirmed that valid interpretations were made.

## Results

The analysis sought patterns across shared experiences, and differences between participants were respected as they contributed to telling unique stories. Despite the diverse sample, six themes emerged. The first three themes were relevant to the issue of identity construction: religion as a public versus private identity, Islam and dressing modestly as expressions of gendered liberation, and sharing a sense of Swiss identity through sameness. The final three were relevant to the issue of belonging: challenging dominant representations, impression management, and religious and cultural identity as psychological strengths.

### Religion as a Public Versus Private Identity

There was a stark contrast between how the women who wore the hijab and how the women who did not were perceived and treated by others. Each woman described her choice to wear or not wear the hijab as a personal decision and did not agree that this choice reflected religiosity. The three women who wore the headscarf recounted experiences of being publicly defined and identified by their religion, which was sometimes essentialized.

“People think just because you’re Muslim, they can’t talk to you about certain things, or some topics are off, [… or] you can’t say your opinion on some things because it’s maybe against Islam or that you’re so—because I’m wearing hijab. Yes, I’m a Muslim woman, but that isn’t all I am.” (Amira)

Amira felt that, as a visibly Muslim woman, people projected their expectations onto her. Having her identity reduced to a single aspect and being expected to have certain opinions was frustrating. In response to perceived stereotyping and identity reduction, Amira drew attention to her identity being multifaceted. Nevertheless, the responsibility to represent Islam was not always framed as a burden:

“I’m really happy to talk about it [Islam]. And so, if people ask questions, I know there is a lot of misinformation, and people don’t know. And if they can come to me and get this information, why not? So, it’s important that I gather this knowledge [about Islam]—but it’s not that it’s a chore like I have to study or something. For me, it was just something that we do.” (Nur)

While the women who wore the hijab recounted experiences of being seen as religious representatives, the women who did not wear the hijab shared a sense of their religious identity being invisible. Although none of the women felt that choosing not to wear the hijab made them less religious, they were aware that their decision affected how others viewed and treated them:

“Questions don’t come at me like whether I’m Muslim or not, because most people would think that being a Muslim woman is someone with the hijab or with the full burka, which is not the case. Because not all Muslim women are wearing hijab, though we are practicing.” (Faheema)

Faheema expressed frustration that other people fail to recognize her as a religious woman because of her choice not to wear the headscarf. In contrast, Hind’s (who also chose not to wear the hijab) preference was to keep her faith private:

“My faith is private, and that is how I like it. I don’t think people view me as a Muslim woman because I don’t wear a hijab. I don’t talk about my religion. I mean, unless it comes up in the conversation and somebody asks me.” (Hind)

Importantly, all the women who did not wear the hijab mentioned a sense of freedom that came with not being recognizably Muslim. The sense of freedom was discussed in terms of not being judged solely as a Muslim woman and feeling less fearful of discrimination and physical attacks. The findings show the detrimental effects of identity reduction during times when the salient identity is under threat.

### Islam and Dressing Modestly as Expressions of Gendered Liberation

The women felt that, far from being oppressive, their faith was a great liberator. Although they differed in their opinions on covering one’s hair and face (one participant was strongly opposed to any type of religious veil), all the women chose to dress modestly and agreed that women were highly valued in Islam:

“The whole idea of women in the Quran is not how it is portrayed now. So, for me—and I’ve never felt that my faith, that Islam has been an oppression upon me. Women are revered in Islam. I don’t feel that Islam dictates to me. I think what Islam does is that it offers you the ideal path. Take, for example, being modest, dressing modestly—I don’t feel that that’s oppressive because it’s my choice.” (Fara)

Similar to five of the women interviewed, Fara stated that misconceptions of female oppression in Islam are partly due to misinterpretation and misunderstanding of Islamic texts. Fara explained that she had studied the Quran extensively and felt strongly that women are highly respected in Islam. Individual choice was cited by five of the participants as an argument against perceived oppression.

Despite their different interpretations of hijab, all the women interviewed opposed the recent “burka ban.” In addition, Zaara cited “Western” gendered norms as restrictive and a barrier to how she would like to express her gender identity:

“We always see it [women’s liberation] from that point, from the Western view. But there are many different points of view. Liberty can be, for you, one thing, and for me, something different. For you, it can be to wear a bikini, and for me, it can be to wear a niqab or a burka or whatever. We should—I think we should stop forcing the Western image of women—of what a woman is.” (Zaara)

Zaara expressed frustration about having a particular ideology of female empowerment forced onto her. She also noted that although this ideal was liberating for some, it was oppressive for others. Furthermore, the interviewees described dressing modestly and wearing the hijab as a form of protection that felt liberating in their everyday lives. This protection was primarily discussed in terms of men’s behavior in public spaces:

“When people see me [with my hijab] or when they don’t know me, it’s always—oh, they step back a bit, and then they try to talk to me. I think that’s a good thing because sometimes, as a woman, people are too much—or like men in general—they are too much in your face. They try to get too close to you.” (Amira)

The gendered guidance in Islam offered the participants the opportunity to express their gender identity in ways that felt empowering and sacred.

### Sharing a Sense of Swiss Identity Through Sameness

Notably, all six participants commented that due to the linguist diversity and cantonal differences, it was challenging to define a collective Swiss identity. However, the women described experiences of identifying with collective Swiss characteristics. These included cultural traits such as being reserved and punctual and having a love of the environment:

“Compared to people in my home country, the people here are a bit more private, a bit quieter, and laid back. They don’t really integrate much with the rest of the people, which is fine. I’m also quite quiet and reserved.” (Faheema)

Faheema highlights some Swiss stereotypes, and although she recognizes that Switzerland is culturally very different from her home country, she notes that these traits are more aligned with her personality.

Fara expressed that she connected to Switzerland through shared values:

“I love the fact that Switzerland cares about, uh, climate change. It has an affinity with me, because, you know, I’m a product of the current world, and clearly, I’m living in a place that aligns with my environmental values.” (Fara)

Nevertheless, although the interviewees acknowledged their similarities with the majority population, these similarities were not enough to encourage a sense of collective identity and belonging for four out of six participants. For example, Hind felt that shared characteristics were superficial:

“Of course, there are similarities, but my religion and my Asianness keep me apart from the Europeans. So, I don’t really belong if you look at it that way.” (Hind)

### Challenging Dominant Representations

All six of the women interviewed were conscious of how Islam is portrayed in the media and were aware of the “oppressed Muslim woman” stereotype. Furthermore, they agreed that there was a distinct lack of positive Muslim women role models presented in the Swiss media. The lack of positive group representation meant that interactions sometimes became moments to dispute stereotypes:

“You know that people know who you are, what you believe, and all the negative representation or the negative narratives that we—Muslims—have in the media. You know, you kind of feel a little bit that you must contrast it, you know, a little bit, somehow—I mean, it’s impossible, but at least to try.” (Zaara)

Zaara challenged these negative perceptions in her everyday life. This sentiment was repeated by five of the six participants. Fara worked as a teacher, and in lessons, she explicitly challenged stereotypes:

“In my role, I’m trying to consciously teach units that are reflecting minorities, one of which is women. As a teacher, I’m trying to break down these stereotypes, starting with my job. I’m lucky that I’m teaching children who are very impressionable.” (Fara)

Sometimes, however, stereotypes were contested unintentionally, as described by Nur:

“I remember once—so, my team leader—it was at the beginning—when, I mean, I started working at my company. And once, I bought some chocolate, like Ferrero Rocher or something. I was like, I’m not opening it now— I’ll open it this evening—because I want me and my husband—we will eat it together. And for me, it was a very normal thing. We always eat sweet things like this together in the evening. And she [the team leader] was like, “Oh!” She said, “Oh, it’s the first time I realized that you’re in love with your husband.” And I was like—yeah! She always thought—before getting to know me—she thought that I would be married to someone—and […] it might have been even an arranged marriage. They assume Muslim people have arranged marriages. Women are oppressed and not happy.” (Nur)

These everyday examples provided the participants with an opportunity to take control of their group narrative by highlighting the difference between how they see themselves and how their group is portrayed in the media.

### Impression Management

Five of the six women interviewed gave examples of adapting their behavior to fit their context. The women who wore the hijab described a shared responsibility that came with being recognizably Muslim. Each of the three women recounted instances where they monitored their behavior in the presence of others because they were aware that wearing the hijab meant that others could judge them as representatives of Islam and not as individuals.

“Even a smile is an act of charity in Islam. Right? So, like, even by smiling, one could say, “Oh, look, Muslims, they are smiling.” And there are people like that! They are kind and nice. Even by doing just this.” (Nur)

Nur recognized how practicing fundamental aspects of Islam could influence others’ opinions of the religion. By adapting her behavior and smiling, she achieved two goals: performing charity and promoting a positive representation of Islam. All three women remarked that this responsibility encouraged them to monitor and positively adapt their behavior. In contrast, other examples of impression management were viewed as negative and restrictive.

“I think if I was to wear the abaya—for example, to go to the mosque— there’s been occasions when I’ve said, “Fara, be brave and go into the mosque,” especially during Ramadan, but the thought of me wearing a hijab and abaya to enter the mosque worries me. Because I think somebody seeing me is going to realize, “Oh, she’s a Muslim, and therefore she must be dangerous, and we need to stay clear from her.” (Fara)

Fara stopped wearing Islamic dress and going to the mosque for fear of being “outed” as a Muslim woman. Thus, she limited her public religious activities to better fit within the community where she lived.

Faheema was also worried about the reaction of others if she were to wear the hijab:

“To wear a hijab now in Switzerland, I still may not be comfortable because people might look at me differently. So that is something that I have to think about if I want to do it somewhere outside of my home country.” (Faheema)

While earlier in the interview, both Faheema and Fara stated it was their personal choice not to wear the headscarf, the above extracts reveal the complexity of their decision as both women describe negotiating personal choice and the fear of Islamophobia. In addition, the extracts demonstrate how Islamophobia can become internalized and restrict identity expression.

### Religious and Cultural Identity as Psychological Strengths

Drawing on personal and religious strengths was one of the most prominent themes. All the interviewees felt at ease discussing their strengths, and these were attributed to their Islamic faith; hardships that they had faced living as a minority group; and innate personality traits. These strengths were identified as coping mechanisms that were critical to the participant's resilience.

“It’s been difficult, but, I mean, that also has to do with my religion […] There’s a reason why this is happening to me, to make me stronger, and I believe […] that it has made me a lot stronger.” (Amira)

Discrimination in Switzerland was often reported as “subtle.” For example, Amira revealed that she had struggled to find employment and felt that this was partly because she wore the hijab. Nevertheless, she viewed this experience through a religious lens and felt that this adversity had given her inner strength.

“I had the feeling that before I reverted to Islam, I was lost in this world. You know, I didn’t have any vision or any idea. I was really lost, and then, since I reverted and got to know this religion […], I feel I have a foundation for the first time in my life. I really enjoy belonging to the Muslim community.” (Zaara)

For Zaara, Islam provided her with a sense of purpose. Despite declaring that she did not physically belong anywhere, she recognized the importance of her faith and the Muslim community in providing her with a sense of belonging. Five of the six participants repeated that belonging to the wider Muslim community provided solace in times when they felt disconnected from their country of residence.

Finally, growing up and experiencing multiple cultural influences or experiencing culturally diverse countries such as Singapore and the UAE was cited as a strength by three of the six participants, as it expanded the participant's worldview.

“I grew up […] in Singapore and, having been exposed to different ethnicities and religions and also learning my religion from a young age, I learned we need to not judge people based on, where they’re from, their color, or religion. So that kind of defines me.” (Faheema)

## Discussion

### Muslim Women’s Construction of Identity in Switzerland

The following themes were important in answering the first research question (How do Muslim women construct identity in Switzerland?): religion as a public versus private identity, Islam and dressing modestly as expressions of gendered liberation, and sharing a sense of Swiss identity through sameness. Through the theme of religion as a public versus private identity, it emerged that the women who wore the hijab were not completely free to choose a salient public identity, as they were judged by their physical appearance. Each of the women who wore the hijab described their decision as personal and did not believe that their choice reflected religiosity. However, their choice of dress did affect how others perceived and treated them. The women who wore the hijab recounted experiences of having their Muslim identity essentialized, which caused frustration for two of the three participants who wore the headscarf. Although the third participant gladly stepped into an ambassador role, it was a role that was given to her at a young age rather than adopted.

These reactions differed from those found by van Es (2019), where, in response to negative stereotyping, Muslim women in the Netherlands participated in self-essentializing. In contrast, the women who wore the hijab in the present study highlighted that, although their Muslim identity was an important aspect of who they are, it was not their only identity. The participants asserted their individuality and emphasized that there was more to them than their outward appearance. Consequently, the women demonstrated the benefits of having multiple identities.

When comparing the findings, it is essential to note the differences between the two studies; van Es’s (2019) study included a much larger participant sample from organized Muslim Women’s groups that actively encourage their members to take on an ambassador role. In contrast, our study included women who weren’t socially connected to one another. In addition, our participants reported that due to Switzerland’s linguistic diversity (which differentiates it from the Netherlands), it was challenging to define a collective “Swiss identity,” which we argue provided greater flexibility in identity construction.

Emphasizing multiple identities could be interpreted as a coping mechanism in response to discrimination. Multiple identities can be used as psychological buffers that protect the individual when an aspect of their sense of self is threatened (Koch & Shepperd, 2004). This coping mechanism may be particularly relevant in the context of the ‘burka ban,’ a political campaign that purposely positioned European and Islamic values as opposing.

In contrast, the three women who chose not to wear the hijab recounted experiences of not being recognized as Muslim. While this caused frustration for one participant, the three participants acknowledged that this also granted them freedom from surveillance. This freedom allowed them to assert their religious identity when they wanted and practice their religion in private. Again, conversely to van Es’s (2019) study, only one of the participants who did not wear the headscarf said they wanted to take on an ambassador role. Nevertheless, she felt restricted in her ability to do so because she was not recognized as a Muslim woman.

Another significant theme in identity construction was Islam and dressing modestly as expressions of gendered liberation. In line with other studies (Allen, 2015; Bouteldja, 2011; van Es, 2019), the participants were aware of the “oppressed Muslim woman” stereotype that they all vehemently opposed. Instead, the women felt that practicing their faith and their decision to dress modestly were expressions of female empowerment. Even though the participants differed in their opinions on the role of the niqab and burka in Islamic practice, they were keen to highlight the contradiction in taking away the freedom of choice to liberate a group with legislation such as the ‘burka ban’.

In contrast to studies such as Bouteldja (2011), the choice to dress modestly and/or wear the hijab was not discussed by the participants as a way to assert their religious identity in reaction to negative representation. Instead, they saw the hijab as offering protection, particularly in relation to men, because it encouraged distance. The women, therefore, expressed part of their gender identity through religious practices. Many of the participants in Bouteldja’s (2011) study wore a full-face veil and thus felt directly affected by the legalization banning their religious garments. In contrast, none of the participants in the present study reported wearing the burka or niqab.

The findings also differ from academic research that focuses on whether Islamic dress makes Muslim women more vulnerable to hate crimes (Allen, 2015; Chakraborti & Zempi, 2012; Zempi, 2020). However, the former studies purposely researched women who had experienced gendered Islamophobia. None of the participants in the present study felt they had experienced “direct” prejudice because of their faith; however, they agreed that discrimination in Switzerland was often “subtle”.

Finally, the women reported sharing a sense of Swiss identity through sameness. *Sameness* was defined as sharing cultural traits and experiences (Brubaker & Cooper, 2000). It is important to note that only two of the six participants felt ‘Swiss’. Cultural traits and collective experiences were cited as more superficial than other identity markers, such as ethnicity and religion.

### Muslim Women’s Sense of Belonging in Switzerland

The following themes were used to answer the second research question (How do Muslim women experience a sense of belonging in Switzerland?): challenging dominant representations, impression management, and religious and cultural identity as psychological strengths. Negative representations of Islam and Muslims were cited as barriers to belonging. Furthermore, although participants cited experiences of feeling valued and accepted—both essential elements of belonging (Hagerty et al., 1992)—these experiences were infrequent.

The participants recognized adverse perceptions of Muslim women and, in response, challenged these representations through education and sometimes unintentionally. Challenging representation has been documented as a known coping mechanism employed by Muslim women in response to negative stereotyping (van Es, 2019). Likewise, educating others is recognized as contributing to society, encouraging a sense of belonging (Hagerty et al., 1992).

Like those in van Es’s (2019) study, the participants remained vigilant during their social interactions. The scrutiny encouraged them to monitor and adjust their behavior to achieve a more positive image. Their actions ranged from smiling to demonstrate that they were not threatening, to concealing their Muslim identity. In some examples, monitoring their behavior was framed as positive because it made them more conscious of their actions. However, in other examples, adjusting behavior to better fit their environment came at the cost of masking an important aspect of their identity.

Muslim women are often portrayed as passive victims (Allen, 2015). In contrast, this study found that the women displayed great resilience and had access to positive coping strategies. Religious and cultural identities that separated the participants from the majority population were framed as strengths. One of the coping mechanisms highlighted was faith, and a relationship with God was integral to everything the participants did. Five of the six participants recognized faith as encouraging a sense of belonging to God and the wider Muslim community. The participants acknowledged that experiencing multiple cultural influences had greatly benefited them by increasing their tolerance and expanding their worldview.

### Limitations and Suggestions for Future Research

Due to time constraints, the sample was small, and recruitment stopped after six women agreed to participate. A larger sample may have been more beneficial. The research was limited to a highly selective sample (i.e., over the age of 18, identified as Muslim, English-speaking, and current Swiss nationals or residents). Future researchers should be aware that allowing participants to speak in their first language may enable them to express themselves more freely.

As non-Muslim women, we were concerned about being outsiders and how this may influence the research. However, we approached our research with open curiosity. This position meant that we did not take the information participants provided for granted and endeavored to discover the meaning behind our participant's experiences. Four of the six participants commented that this factor aided their disclosure as they did not feel pressured to live up to any religious or cultural standards.

### Conclusion

The Muslim women interviewed faced challenges in forming positive Muslim identities and experiencing a sense of belonging in Switzerland. Nevertheless, challenges were met with great strength and resilience. The women took control of their narratives by challenging negative representations, expressing their gendered identity in ways that contradicted the dominant culture, and emphasizing their multiple identities. However, achieving both a positive Muslim identity and a sense of belonging in Switzerland sometimes came at the cost of concealing their religious identity and monitoring their behavior.

### Implications for Counseling Practice

Based on previous research and the results, the following guidelines are proposed for counselors in this context. First, non-Muslim counselors are encouraged to question their own assumptions and biases. Counselors need to be aware of the likelihood of negative depictions of Islam and Muslim women being internalized not only by themselves but possibly also by their Muslim clients. Second, counselors need to remain open to exploring the importance of religion for their clients. Not integrating religion into therapy could strengthen Muslim women’s beliefs that parts of their identity should be hidden. Third, counselors need to be careful not to solely define Muslim women by their religion. Exploring intersections of various identities is vital in understanding each client’s unique worldview and in helping to counter any negative experiences of essentialization. Finally, counselors are encouraged to adopt a strengths-based approach. Highlighting resources can emphasize the resilience of clients who have experienced discrimination.
